# The counter regulatory axis of the renin angiotensin system in the brain and ischaemic stroke: Insight from preclinical stroke studies and therapeutic potential

**DOI:** 10.1016/j.cellsig.2020.109809

**Published:** 2020-12

**Authors:** Aisling McFall, Stuart A. Nicklin, Lorraine M. Work

**Affiliations:** Institute of Cardiovascular & Medical Sciences, College of Medical, Veterinary & Life Sciences, University of Glasgow, Glasgow, UK

**Keywords:** Renin angiotensin system, Ischaemic stroke, AT_2_R, Mas receptor, Ang-(1–7), Ang-(1–9), C21, AD, Alzheimer's disease, Aβ), amyloid β, Ang I), angiotensin I, Ang II), angiotensin II, Ang-(1–7)), angiotensin-(1–7), Ang-(1–9)), angiotensin-(1–9), ACE), angiotensin converting enzyme, ACE2), angiotensin converting enzyme 2, ACEi), angiotensin converting enzyme inhibitor, ARB), angiotensin receptor blocker, AT_1_R), angiotensin II type 1 receptor, AT_2_R), angiotensin II type 2 receptor, BBB), blood brain barrier, BP), blood pressure, B_2_R), bradykinin type 2 receptor, BDNF), brain derived neurotrophic factor, CBF), cerebral blood flow, C21), compound 21, DIZE), diminazene aceturate, eNOS), endothelial nitric oxide synthase, ET-1), endothelin-1, ERK 1/2), extracellular signal related kinase 1/2, GPCR), G-protein coupled receptor, MrgD), Mas related GPCR type D, MAPKs), mitogen activated protein kinases, NOX), NAD(*P*)H oxidases, nNOS), neuronal nitric oxide synthase, NO), nitric oxide, pMCAO), permanent middle cerebral artery occlusion, ROS), reactive oxygen species, RAS), renin angiotensin system, SHR), spontaneously hypertensive rat, tPA), tissue plasminogen activator, tMCAO), transient middle cerebral artery occlusion

## Abstract

Stroke is the 2nd leading cause of death worldwide and the leading cause of physical disability and cognitive issues. Although we have made progress in certain aspects of stroke treatment, the consequences remain substantial and new treatments are needed. Hypertension has long been recognised as a major risk factor for stroke, both haemorrhagic and ischaemic. The renin angiotensin system (RAS) plays a key role in blood pressure regulation and this, plus local expression and signalling of RAS in the brain, both support the potential for targeting this axis therapeutically in the setting of stroke. While historically, focus has been on suppressing classical RAS signalling through the angiotensin type 1 receptor (AT_1_R), the identification of a counter-regulatory axis of the RAS signalling via the angiotensin type 2 receptor (AT_2_R) and Mas receptor has renewed interest in targeting the RAS. This review describes RAS signalling in the brain and the potential of targeting the Mas receptor and AT_2_R in preclinical models of ischaemic stroke. The animal and experimental models, and the route and timing of intervention, are considered from a translational perspective.

## Highlights

• There is an unmet need for novel therapies for treating ischaemic stroke.
• The brain renin angiotensin system (RAS) provides a target for stroke treatment.
• Cellular signalling via the counter regulatory RAS axis may aid brain repair.
• Stimulating counter regulatory RAS is beneficial in preclinical stroke models.
• Timing and route of delivery require to be addressed for clinical translation.

## 1 Introduction

Stroke is a leading cause of death and disability worldwide [1], and can result in the development of dementia in 30% of cases [2,3], yet treatment options for this condition are limited. Stroke can be caused by a ruptured cerebral blood vessel, known as haemorrhagic stroke, or more commonly by the blockage of a blood vessel within the brain, known as ischaemic stroke [4]. Starvation of the brain tissue of oxygen and glucose during ischaemia results in a pathophysiological cascade of damage consisting of ionic dysregulation, excitotoxicity, oxidative stress and inflammation [5]. Recanalization of the vessel, either pharmacologically using a clot busting drug known as tissue plasminogen activator (tPA) or by mechanical clot removal known as thrombectomy remain the only available clinical interventions. Different aspects of stroke pathophysiology have been previously targeted as neuroprotective strategies [6], but so far, aside from improvements achieved utilising thrombectomy [[7], [8], [9]] there has been no progress towards improving patient outcome following ischaemic stroke. It is recognised that reperfusion paradoxically results in further injury by reperfusion injury [10] but the benefit achieved through recanalization outweighs the cost associated with failure to restore blood flow. There is an ongoing quest to develop neuroprotective strategies to increase tissue salvage and improve functional outcome for patients post stroke and the hope is that the efforts of the preclinical stroke community to improve methodological rigor in experimental stroke research [[11], [12], [13]] will bring a novel neuroprotectant to fruition.

The renin angiotensin system (RAS) is a physiological system that maintains cardiovascular homeostasis through maintenance of arterial blood pressure (BP). Classical RAS signalling mediated by Angiotensin II (Ang II) via the Angiotensin II type 1 receptor (AT_1_R) increases systemic BP; however it also has further effects on a tissue level that are implicated in disease [[14], [15], [16]]. Hypertension is the key modifiable risk factor for stroke [17], systemic RAS blockade, with ACE inhibitors (ACEi) or Ang II type 1 receptor (AT_1_R) antagonists (angiotensin receptor blockers; ARBs), is a common therapy for treating hypertension [18] and with efficient BP control the risk of stroke is reduced [19,20]. However, the benefits of modulation of BP acutely post-stroke remains controversial [21]. Evidence of the existence of local expression and signalling of RAS within the brain may provide a potential therapeutic target for neurological or cerebrovascular disorders including ischaemic stroke, although whether this will be independent of BP modulation will be dependent on many factors including dose and route of delivery. This review will outline RAS receptor signalling and summarise the current available evidence for the potential benefit of counter-regulatory RAS receptor agonism in ischaemic stroke.

## 2 The Renin Angiotensin System

**Fig. 1:**
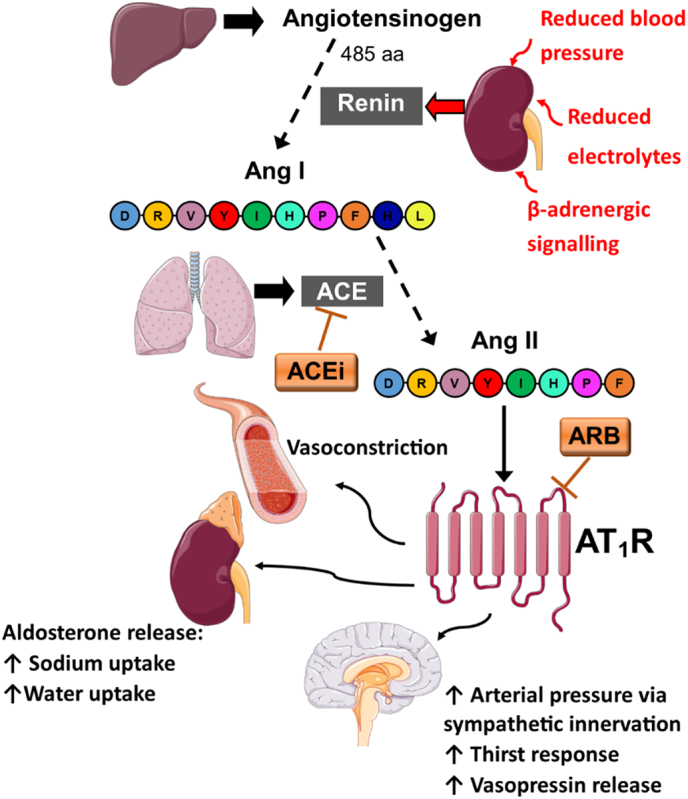
**The classical renin angiotensin system.** The protein angiotensinogen is constitutively released from the liver into the circulation [22]. In response to detection of reduced BP, reduced electrolytes or sympathetic innervation in the kidney [23], the enzyme renin is secreted from the kidney (red) which cleaves angiotensinogen to the decapeptide angiotensin I (Ang I). Angiotensin converting enzyme (ACE) is present on the endothelial wall of blood vessels in particular in the lung [24,25]. ACE cleaves Ang I to the octapeptide Ang II, which acts upon the angiotensin II type 1 receptor (AT_1_R) to increase BP and blood volume by vasoconstriction of blood vessel [26], stimulating aldosterone release from the adrenal gland (on top of the kidney) and hence increasing sodium and water uptake [27], and actions on the brain increasing arterial pressure by sympathetic innervation or to increase the thirst response and release vasopressin to increase water uptake [28,29]. ACE inhibitors (ACEi) or angiotensin receptor blockers (ARB) (orange) are BP lowering medications which block these elements of the RAS to prevent increased BP. Letters in peptides indicate the amino acid sequence. (For interpretation of the references to colour in this figure legend, the reader is referred to the web version of this article.)

In the classical systemic RAS, the biologically active peptide, Ang II, is produced through the actions of the enzymes renin and angiotensin converting enzyme (ACE) and exerts its effects primarily through the AT_1_R causing increased BP via vasoconstriction, increased water and sodium uptake in the kidney directly and via aldosterone and vasopressin release, and stimulation of the thirst response. (Fig. 1 and associated references).

**Fig. 2:**
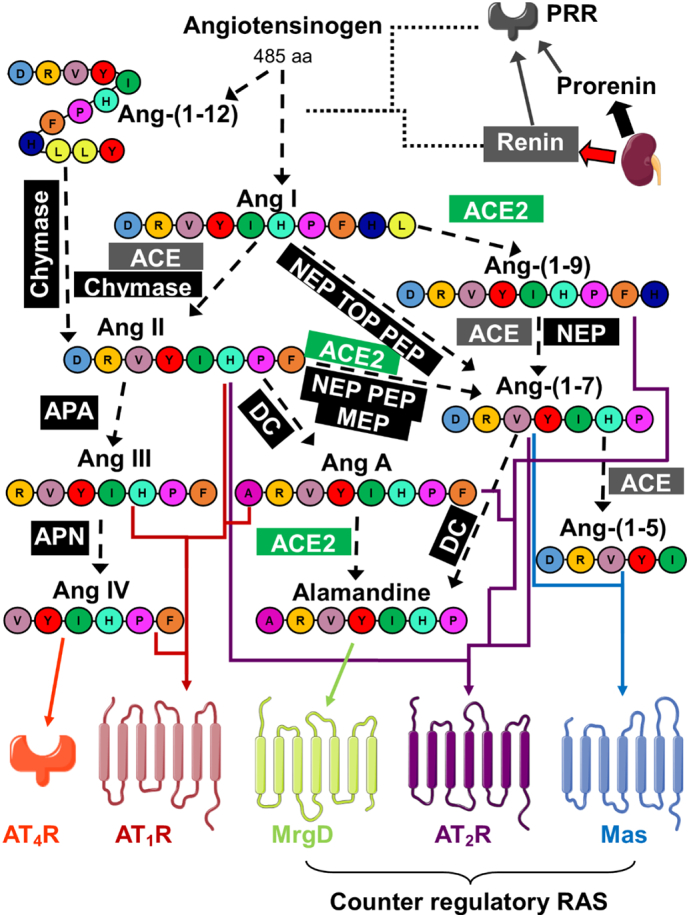
The extended RAS and counter-regulatory axis. Diagram illustrating the additional discoveries in the RAS. Prorenin is constitutively secreted from the kidney (black block arrow) while cleaved renin is secreted in response to stimuli (red block arrow) [31]. Renin acts directly on angiotensinogen to cleave it to Ang I, while both prorenin and renin can bind to the (pro)renin receptor (PRR) allowing increased cleavage activity of angiotensinogen [32]. The classic and counter-regulatory enzymes for angiotensin peptide cleavage, angiotensin converting enzyme (ACE) (grey) [33] and ACE2 (green) [34] are shown along with additional enzymes which result in peptide cleavage (black) [[35], [36], [37], [38], [39], [40], [41], [42], [43], [44]]. Dashed lines represent cleavage while coloured arrows indicate action of the peptide upon the colour coded receptors. Ang-(1–5), Ang III, Ang IV, Ang-(1–7) and Ang-(1–9) are all active peptides in the RAS [[45], [46], [47], [48], [49]]. Ang A shows a reduced vasoconstrictive effect through AT_1_R compared to Ang II and also acts upon the AT_2_R [50]. The angiotensin II type 2 receptor (AT_2_R), Mas receptor and Mas related GPCR type D (MrgD) receptor form the counter-regulatory axis of the RAS, opposing the signalling effects of AT_1_R [30]. Furthermore, AT_1_R and Mas [51], AT_1_R and AT_2_R [52], and AT_2_R and Mas [53] have been found to form signalling heterodimers. AT_4_R is not a GPCR like the other angiotensin receptors, but an enzyme, insulin-regulated membrane aminopeptidase (IRAP) [54]. Abbreviations: NEP, neprilysin; TOP, thimet oligopeptidase; PEP, prolyl-endopeptidases; MEP, metalloendopeptidases; APA, aminopeptidase A; APN, aminopeptidase N; DC, decarboxylase enzyme. Letters in peptides indicate the amino acid sequence. (For interpretation of the references to colour in this figure legend, the reader is referred to the web version of this article.)

A counter-regulatory axis of this system exists [30], consisting of an alternative enzyme, ACE2, which produces the peptides, Ang-(1–9) and Ang-(1–7), and alternative receptors for these to act through, the Ang II type 2 receptor (AT_2_R) and Mas receptor respectively. This produces antagonistic effects to AT_1_R signalling. This is a simplistic summary of the RAS and counter-regulatory RAS but realistically the system is much more complex consisting of various other enzymes, peptides and receptors as outlined in Fig. 2.

## 3 The RAS in the brain

The existence of RAS signalling in the brain has long been known; indeed early research on Ang II and Ang-(1–7) implied direct actions in the brain [[55], [56], [57]]. The central/brain RAS plays a role in the control of systemic BP mediated by sympathetic innervation [58], vasopressin release [59] and increased thirst [60], and inhibition of the central RAS, by intracerebroventricular injection of the prototype ARB saralasin, has been shown to reduce hypertension [61]. Although the majority of RAS research focusses on Ang II, it has been shown that conversion of Ang II to Ang III in the brain is responsible for the pressor response, where Ang II was ineffective when its conversion to Ang II was blocked with an aminopeptidase A (APA) inhibitor (Fig. 2), and thus Ang III may be the active peptide of classical RAS signalling in the central RAS [62]. Indeed, APA inhibitors are in clinical development for the treatment of hypertension (reviewed in [63]). In the brain, angiotensin immunoreactive nerve fibers have been mapped to demonstrate that they originate in circumventricular organs (CVO) (such as the subfornical organ, organum vasculosum of the lamina terminalis and area postrema). The CVO are areas of the brain which have no blood brain barrier (BBB) and therefore can directly interact with the circulation and circulating RAS peptides. The angiotensinergic neurons project to the paraventricular nucleus, supraoptic nucleus and the nucleus of the solitary tract [64,65] which are all neural circuits involved in fluid regulation, vasopressin release, sympathetic innervation and thirst response [66].

Furthermore, expression of the RAS components has been demonstrated within the brain, with renin activity demonstrated in the brain independent of circulating renin [67], angiotensinogen mRNA detected across the rat brain [68] and in both astrocytes [69] and neurons [70], ACE detected by radioligand binding in various regions of the human brain [71], and detection of Ang II and other angiotensin peptides (including Ang-(1–9) and Ang-(1–7)) in rat and sheep brains [72]. Following the discovery of ACE2, its expression was also confirmed within the brain at both a mRNA and protein level [73], and expression of the RAS receptors, AT_1_R, AT_2_R and Mas receptor, in the brain have been demonstrated by numerous studies [[74], [75], [76], [77], [78], [79], [80], [81], [82], [83]]. Recently, however, the existence of a locally expressed brain RAS has been questioned when van Thiel *et al*. demonstrated that, despite angiotensinogen mRNA expression in mouse brains, angiotensinogen protein was not detected, and perfusion of brains before analysis resulted in a marked reduction in renin activity, suggesting that the detected renin in brain tissues may just be circulating renin trapped within blood vessels in the tissues [84]. In contrast, using a newly developed microanalytical assay, coupling a laboratory-built capillary electrophoresis nano-electrospray ionization platform to a high-resolution mass spectrometer, several of the Ang peptides, including Ang I, Ang II, Ang-(1–7) and Ang-(1–9), were detected in the subfornical organ and the paraventricular nucleus of the hypothalamus in mice [85].

## 4 RAS receptor signalling

### 4.1 AT_1_R

The AT_1_R is a G-protein coupled receptor (GPCR) expressed throughout tissues of the body. In humans, a single gene exists for the receptor, however in rodents two pharmacologically indistinguishable isoforms exist, AT_1A_R and AT_1B_R [86]. The tissue expression of these isoforms varies, with AT_1A_R being the predominant isoform in all tissues, including brain, vasculature, lung and liver, except the adrenal and pituitary gland where AT_1B_R is the predominant isoform [87]. AT_1_R is regulated at a transcriptional level by being downregulated by Ang II [88] and can also be modified by numerous other factors such as insulin, glucose, estrogen, chemokines, nitric oxide (NO), reactive oxygen species (ROS) or low-density lipoprotein (LDL) cholesterol [89] with insulin, for example, increasing AT_1_R expression in vascular smooth muscle cells [90] and estrogen [91] and NO [92] leading to a down-regulation of AT_1_R in the hypothalmus or in neurons respectively. AT_1_R is also regulated by desensitisation to further activation by being rapidly internalised [93] or phosphorylated [94,95] upon activation, or, as is a common feature of GPCR signalling [96], AT_1_R can also be regulated by the formation of heterodimers with other receptors such as the bradykinin receptor (B_2_R) [97], the β-adrenergic receptor [98], the Mas receptor [51] or the AT_2_R [52].

**Fig. 3:**
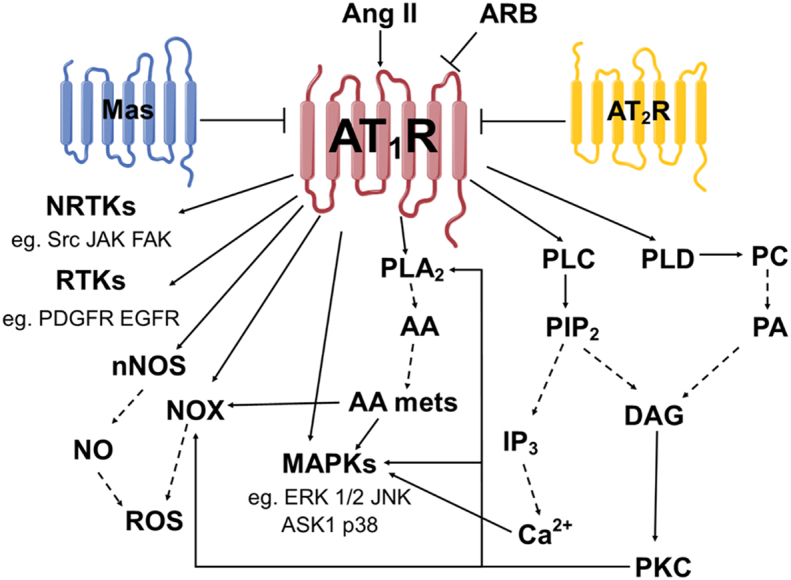
**AT**_**1**_**R signalling.** Diagram depicting summary of intracellular signalling cascades induced by angiotensin II type 1 receptor (AT_1_R) activation by Ang II, or blocked by angiotensin receptor blockers (ARBs) or heterodimerisation with angiotensin II type 2 receptor (AT_2_R) or Mas receptor. Arrows indicate activation while dashed line arrows indicate production or cleavage to form a product. Signalling information obtained from [16,51,[121], [122], [123],^52^,^107^,[115], [116], [117], [118], [119], [120]]. Abbreviations: AA mets, arachidonic acid metabolites; AA, arachidonic acid; ASK1, apoptosis signal regulating kinase 1; DAG, diacylglycerol; ERK 1/2, extracellular signal related kinase 1/2; FAK, focal adhesion kinase; IP3, inositol trisphosphate; JAK, Janus kinase; JNK, c-Jun N-terminal kinase; MAPKs, mitogen activated protein kinases; NOX, NAD(*P*)H oxidases; nNOS; neuronal nitric oxide synthacse; NRTKs, non-receptor tyrosine kinases; p38, p38 MAPK; PA, phosphatidic acid; PC, phosphatidylcholine; PDGFR, platelet derived growth factor receptor; PIP2, phosphatidylinositol 4,5-bisphosphate; PKC, protein kinase C; PLA2, phospholipase A2; PLC, phospholipase C; PLD, phospholipase D; ROS, reactive oxygen species; RTKs, receptor tyrosine kinases.

Downstream signalling from Ang II at the AT_1_R involves activation of phospholipase C (PLC), phospholipase D (PLD) and phospholipase A_2_ (PLA_2_) along with activation of mitogen-activated protein kinases (MAPKs) and NADPH oxidases (NOXs), non-receptor tyrosine kinases (NRTKs) or receptor tyrosine kinases (RTKs) (Fig. 3 and associated references). In the systemic circulation, these signalling events result in contraction of vascular smooth muscle cells but also reactive oxygen species (ROS) production, inflammation, apoptosis, proliferation, protein synthesis, cell growth and migration influencing cell survival and pathological effects such as hypertrophy and fibrosis [16,99]. Indeed, much of the research on downstream signalling from AT_1_R has been conducted in vascular or cardiac cells, however Ang II mediated effects in the brain have also been attributed to some of these signalling pathways. For example, AT_1_R stimulation with Ang II in neuronal cultures results in activation of MAPKs [100] while AT_1_R blockade results in the reduction of MAPK activation, with MAPK activation being associated with microglial or astrocyte activation [101]. Within the brain, classical RAS signalling through AT_1_R leads to: vasopressin release [59] and suppression of the baroreflex, which has been shown to be mediated via PLC activation [102]**;** a switch towards the proinflammatory microglia phenotype with increased levels of proinflammatory cytokines such as tissue necrosis factor α (TNFα) or interleukin-1β (IL-1β) [103]; increased astrogliosis [104]; or in neurons, increased mitochondria-dependent apoptosis [105], increased production of ROS leading to apoptosis [106,107] or autophagy activation contributing to Ang II induced apoptosis via the AT_1_R [108]. Ang II induces ROS production in neurons via NOX activation which has been demonstrated to be mediated by protein kinase C (PKC) [109], but neuronal NO synthase (nNOS) is also upregulated by Ang II stimulation of neurons, contributing to oxidative stress and subsequent apoptosis induction [107]. PKC mediated activation of NOX leading to ROS production also results in microglial activation via activation of Rho-kinase by NFκB which additionally feeds back to further upregulate AT_1_R expression [110,111]**.** There is also evidence to suggest that Ang II stimulation of AT_1_R inhibits iron uptake in neurons [112,113] and iron metabolism dysregulation is linked to neurodegeneration [114].

### 4.2 Mas receptor

The Mas receptor is a GPCR coded for by a gene originally identified as a proto-oncogene [124] but suggested early to code for an angiotensin receptor [125]. Like AT_1_R and other GPCRs, upon activation by Ang-(1–7), the Mas receptor is internalised and therefore desensitised to further activation [126]. Additionally, the Mas receptor can form heterodimers with other GPCRs, for example with AT_2_R which is necessary for signalling in some cell types [53], or with AT_1_R causing an inhibitory, regulatory effect on AT_1_R [51]. Ang-(1–7) is the heptapeptide product of ACE2 cleavage of Ang II or of ACE cleavage of Ang-(1–9) [34,39] (Fig. 2), which shows counter-regulatory effects to AT_1_R signalling [46] and mediates its effects through the Mas receptor [127]; although there have also been reports of effects mediated via the AT_2_R [128]. AVE 0991 is a nonpeptide, commercially available agonist at the Mas receptor, originally characterised in endothelial cells [129] while conversely, A779 acts as an antagonist of the Mas receptor [130].

**Fig. 4:**
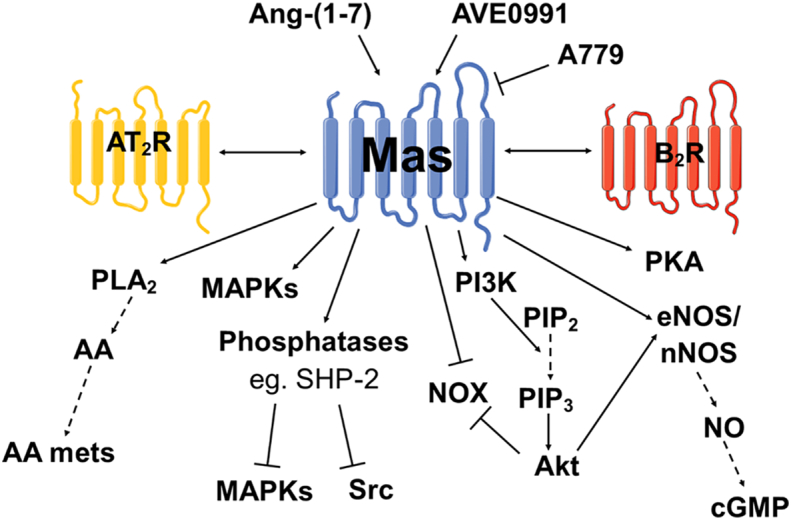
Mas receptor signalling. Diagram depicting summary of intracellular signalling cascades induced by Mas receptor (Mas receptor) activation. Agonists and antagonists are also shown along with receptors with which functional heterodimerisation can occur (AT_2_R and the bradykinin 2 receptor (B_2_R)). Arrows indicate activation while dashed line arrows indicate production or cleavage to form a product. Signalling information obtained from [127,131,166,167,[132], [133], [134],^137^,^140^,^156^,^164^,^165^]. Abbreviations: AA mets, arachidonic acid metabolites; AA, arachidonic acid; Akt, protein kinase B; cGMP, cyclic guanidine monophosphate; eNOS, endothelial nitric oxide synthase; MAPKs, mitogen activated protein kinases; NO, nitric oxide; PI3K, phosphoinositide-3-kinase; PIP_2_, phosphatidylinositol 4,5-bisphosphate; PIP_3_, phosphatidylinositol 3,4,5-trisphosphate; PKA: protein kinase A; PLA_2_, phospholipase A_2_; SHP-2, SH2 domain-containing protein tyrosine phosphatase-2.

Downstream signalling following Mas receptor activation by Ang-(1–7) includes activation of the phosphoinositide-3-kinase (PI3K) signalling pathway and endothelial NO synthase (eNOS) activation along with activation of tyrosine phosphatases, PLA_2_, protein kinase A (PKA) or MAPKs (Fig. 4 and associated references). The production of NO and arachidonic acid (AA) metabolites causes vasodilation in opposition to Ang II AT_1_R mediated vasoconstriction [127,131,132], while inhibition of MAPKs or Src attenuates remodelling in heart [[133], [134], [135]] or vasculature [136]. Conversely, however, the activation of MAPKs, such as ERK1/2 or p38 MAPK, by Mas receptor activation has been implicated in vasodilation and angiogenesis [137].

Specifically in the brain, Ang-(1–7)/Mas has been shown to modulate central cardiovascular regulation with a BP and heart rate lowering effect when Ang-(1–7) was injected into the nucleus of the solitary tract (NTS) region within the dorsal medulla of rats [57]. This hypotensive effect is mediated by NO production [138,139], or more specifically the bradykinin dependent NO pathway of Mas signalling stimulating cyclic GMP (cGMP) and cGMP-dependent protein kinase (PKG), resulting in reduced norepinephrine release and reduced sympathetic innervation [140]. Conversely, however, central Ang-(1–7) administration into the ventrolateral medulla (VLM) of rats, either the rostral VLM (RVLM) [[141], [142], [143]] or the caudal pressor area (CPA) of the VLM [141,143], or the RVLM, but not caudal VLM, of rabbits [144] resulted in an increase in BP. Additionally, Ang-(1–7) can induce vasopressin release, although this may not be mediated by the Mas receptor [145]. It has also been suggested that Ang-(1–7)/Mas signalling in astrocytes rather than neurons may play an important role in cardiovascular regulation [146]. Taken together, these studies may indicate divergent effects of Ang-(1–7) depending on the brain region or angiotensin receptor expression in that area.

Aside from cardiovascular regulation however, Ang-(1–7) and Mas receptor signalling have demonstrated specific effects on brain cells such as: reduced nuclear and mitochondrial superoxide production [147]; improved neuronal survival and reduced oxidative stress in the brain mediated via PKA [148], or improved survival and reduced levels of ROS in rotenone induced injury of cultured neurons mediated by increased antioxidant activity and reduced NOX activity [149]; reduced microglial activation and astrogliosis allowing for improved neuronal density following traumatic brain injury [150]; reduced inflammation in astrocytes mediated by the MAPK inhibition signalling pathway [151]; reduced Shiga toxin 2 (Stx2) induced neuronal, astrocyte and oligodendrocyte damage [152]; or a switch to the anti-inflammatory M2 microglia phenotype with a reduction in pro-inflammatory cytokines (TNFα and IL1β) and increased levels of anti-inflammatory IL-10 [153]. Moreover, central Ang-(1–7) administration has demonstrated an increase in bradykinin levels and expression of bradykinin receptors [154] which may itself have neuroprotective effects [155]; or protection against cerebral endothelial cell dysfunction and oxidative stress via NOX inhibition and the PI3K/NO signalling pathway [156]. Additionally, Mas receptor signalling pathways (Fig. 4) have been demonstrated specifically in neurons with increased NO, AA release, MAPK activation and protein kinase B (PKB/Akt) activation with Ang-(1–7) treatment [157], or the downregulation of p38 MAPK and NOX coupled with the activation of PI3K and PKB/Akt resulting in increased levels of brain derived neurotrophic factor (BDNF), a neuronal survival promoting protein [158].

Finally, Ang-(1–7)/Mas signalling has also been implicated in learning and memory, with Ang-(1–7) increasing long-term potentiation in the hippocampus or amygdala via NO production [159,160], improving cognitive function in diabetic rats [161], or ACE2 activation improving cognition, coupled with reduced neuronal apoptosis, reduced inflammation and increased levels of BDNF, mediated via PI3K, in an Alzheimer's disease (AD) model [162]. Moreover Mas activation has demonstrated reduced anxiety like behaviour [163].

### 4.3 AT_2_R

The AT_2_R shares 34% homology with AT_1_R with a similar seven transmembrane GPCR structure [168]. Its expression is highest in fetal tissues and declines after birth [169] although more recently this concept was challenged with a study showing higher levels of AT_2_R protein in brainstem, liver and kidney in adult rats compared to fetal or neonatal rats [170]. The gene coding for AT_2_R has been mapped to the X chromosome [171] and studies in rodents have indicated a higher expression of AT_2_R in females compared to males due to upregulation by estrogen in the brain and heart [172,173], however, others have recently reported no overall sex related differences in AT_2_R, or AT_1_R, expression in the mouse brain [82]. Other regulators of AT_2_R expression include glucocorticoids and cytokines [174] or specifically, the nuclear protein poly(ADP-ribose) polymerase-1 (PARP1) [175] or transcription factor promyelocytic zinc finger protein (PLZF) [176] which regulate AT_2_R transcription. Further, the AT_2_R binding protein (ATBP)/AT_2_R interacting protein (ATIP) interacts with the C terminus of the receptor to promote its expression on the cell membrane [177,178]. Various other angiotensin peptides are reported to be ligands at the AT_2_R, namely Ang III, Ang IV, Ang-(1–7) and Ang-(1–9), although the affinity for Ang II is the highest [47,179]. Additionally, peptide (CGP42112 [180], β-Pro [7] Ang III [181]) and non-peptide (Compound 21, C21 [182]) agonists of AT_2_R have been developed which have further aided the understanding of AT_2_R signalling.

Unlike the AT_1_R and other GPCRs, the AT_2_R does not display internalization and desensitisation in response to ligand binding [183] and can therefore induce long term signalling effects. However, similar to other GPCRs, AT_2_R can form functional heterodimers for example with Mas receptor [53] or the B_2_R [184], and acts as an antagonist of AT_1_R by forming a heterodimer with this receptor independent of AT_2_R signalling [52]. In addition, AT_2_R forms homodimers causing constitutive activation without Ang II stimulation [185].

**Fig. 5:**
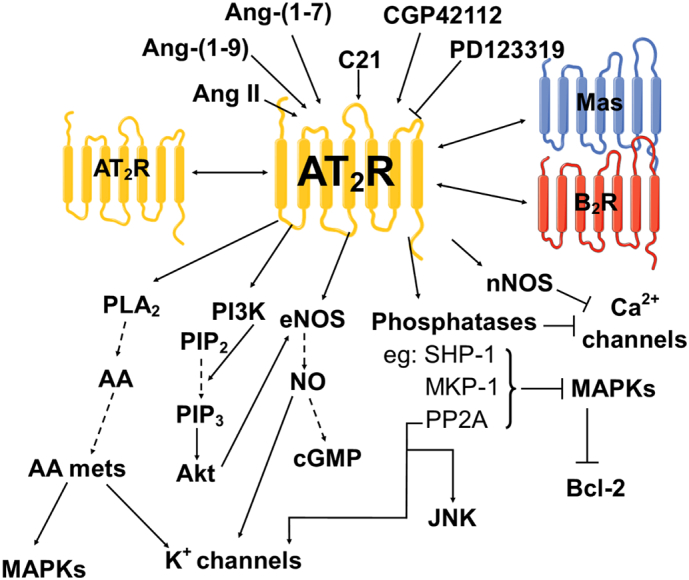
AT_2_R signalling. Diagram depicting summary of intracellular signalling cascades induced by angiotensin II type 2 receptor (AT_2_R) activation. Agonists and antagonists are also shown along with receptors with which functional heterodimerisation or homodimerization can occur. Arrows indicate activation while dashed line arrows indicate production or cleavage to form a product. Signalling information obtained from [53,100,219,[221], [222], [223], [224], [225], [226], [227],^128^,^174^,^186^,^187^,[203], [204], [205], [206]]. Abbreviations: AA mets, arachidonic acid metabolites; AA, arachidonic acid; Bcl-2, B-cell lymphoma 2; cGMP, cyclic guanidine monophosphate; eNOS, endothelial nitric oxide synthase; JNK; c-Jun N-terminal kinase; MAPKs, mitogen activated protein kinases; MKP-1, MAPK phosphatase-1; NO, nitric oxide; PLA_2_, phospholipase A_2_; PP2A, protein phosphatase 2A; SHP-1, Src homology region 2 domain-containing phosphatase.

AT_2_R signalling is mediated through activation of protein phosphatases, or, similar to Mas receptor signalling, regulation of NO-cGMP, and activation of PLA_2_ leading to systemic effects opposing AT_1_R signalling such as vasodilation or anti-fibrotic or anti-hypertrophic effects [30] (Fig. 5 and associated references). Additionally, however, inhibition of MAPK activation due to phosphatase activity via AT_2_R signalling can result in apoptosis induction and this has been demonstrated in neuronal cells [186,187]. The induction of apoptosis by AT_2_R signalling occurs in tumor cells, thus implicating this receptor in possible cancer treatments [188,189], and also in Chinese hamster ovary and vascular smooth muscle cells [190]. In primary neuronal cultures, AT_2_R contributed to apoptosis induction only when in combination with other insults such as zinc treatment [191] or ultraviolet light exposure [187] suggesting additional complexities to AT_2_R signalling depending on the environment. Conversely, cell death was reduced *in vitro* in neurons subjected to glucose deprivation treated with the AT_2_R agonist, CGP42112 but not C21 [192]. Interestingly, however, *in vivo* delivery of CGP42112 (ip) [192] or C21 (daily ip injections) [193] resulted in reduced numbers of apoptotic neurons after experimental stroke in mice. Combined, these studies demonstrate AT_2_R stimulation may exert a beneficial effect through both apoptotic and anti-apoptotic mechanisms, perhaps dependent on the cell type affected or timing in terms of the balance between neuro-injury and neurorepair.

Other effects of AT_2_R signalling in the brain are reduced ROS production [194], improved neuronal cell survival [195,196], a switch to the anti-inflammatory M2 microglia phenotype [110], and a reduction in the pro-inflammatory cytokine, TNFα, and increased levels of anti-inflammatory IL-10 [197,198]. Moreover, AT_2_R signalling in brain cells has been shown to directly oppose AT_1_R signalling; for example reducing Ang II induced NOX activity and subsequent ROS production in neuronal cultures [199], or activation of protein phosphatase 2A (PP2A) causing inhibition of PKC and the subsequent NOX activation and ROS production which would cause microglial activation [110]. In addition, the phosphatase mediated inhibition of MAPK signalling pathway of AT_2_R, namely PP2A inhibition of ERK1/2 activity, has been confirmed in neuronal cultures [100,200]. Interestingly, AT_2_R stimulation also modulates vasopressin release in the brain via synapse interaction of AT_2_R positive neurons with vasopressin secreting neurons, counteracting Ang II induced vasopressin release by AT_1_R [201].

Further effects of AT_2_R signalling in neurons include the modulation of neuronal excitability, through reduction of T-type calcium channel currents via phosphotyrosine phosphatase activity [202,203]. or increased potassium channel currents via PLA_2_, PP2A activation or NO production [[204], [205], [206]]; induction of neurite outgrowth and differentiation via NO production [[207], [208], [209], [210]] which may be via the PI3K/Akt pathway [211] or increased MAPK (ERK1/2) activity [209,212,213]; increased neuronal migration via PP2A activation [214]; or induction of neurogenesis and neural stem cell proliferation via Akt activation, ERK activation and modulation of potassium channels [215]. Conversely however, other neuronal cell lines have demonstrated reduced proliferation with AT_2_R stimulation [213].

The demonstration of local effects of both AT_2_R and Mas receptor signalling in the brain has led to the exploration of targeting the RAS for many neurological disorders, for example Parkinson's disease [216], depression [217], Alzheimer's Disease (AD) [218], cognitive impairment [219] or as reviewed here and elsewhere, the cerebrovascular disorder, stroke [220].

## 5 The classical RAS in stroke

Blockade of the systemic RAS is routinely used for the treatment of hypertension through the use of ARBs and ACEis [18] and therefore this allows for the analysis of the effect of these systemic drugs on stroke outcome by acute BP modulation or risk through long-term BP control. In the LIFE trial, treatment for hypertension with the ARB losartan resulted in 25% fewer strokes than with the β1 receptor antagonist (β-blocker) atenolol despite similar BP reductions [228], while the MOSES trial demonstrated fewer cerebrovascular and cardiovascular events over a 2.5 year follow-up period when patients who had suffered a stroke in the past two years were treated with the ARB eprosartan compared to the calcium channel blocker nitrendipine, despite similar BP reductions with both drugs [229]. In a randomized controlled trial comparing treatment with the ARB candesartan immediately following stroke or delayed until seven days later (ACCESS trial) there was no significant difference in functional outcome at three months, however early ARB treatment did significantly reduce the number of cardiovascular events [230]. An observational study of French stroke cohorts demonstrated no benefit of on-going ACEi or ARB treatment on stroke outcomes at 3 months [231]. However, in contrast to those studied described above, these patients also received thrombolysis with rt-PA. A meta-analysis of 26 clinical trials indicated that ARBs and ACEis offer no BP independent risk reduction for stroke [232].

Despite these varied results in clinical trials, there remains significant evidence that the brain RAS plays a role in ischaemic stroke pathophysiology and may offer potential as a therapeutic target. In experimental stroke models, intracerebroventricular infusion of an ARB resulted in beneficial outcomes of improved neurological score or decreased infarct size following transient middle cerebral artery occlusion (tMCAO), a commonly used model of ischaemia-reperfusion stroke injury, but did not lower the systemic BP due to the low dose utilised [233,234]. The dose was chosen to allow any BP-independent effects of the ARB to be identified and to avoid worsening outcome through aggressive BP lowering in the acute period after stroke [233,234]. Similarly, with systemic administration of an ARB dose too low to exert a BP lowering effect, pre- or post-tMCAO, reduced infarct volumes and improved functional outcomes were observed [[235], [236], [237], [238]]. The ACEi, ramipril, however, demonstrated no neuroprotective effects with systemic delivery prior to tMCAO [235]. Furthermore, vaccination with an anti-Ang II antibody demonstrated neuroprotective effects with reduced infarct volume following permanent occlusion of the middle cerebral artery (pMCAO), another commonly used stroke animal model [239]. Although the antibody did not appear to penetrate the brain tissue of control rats, it could penetrate the ischaemic lesion to block AT_1_R signalling in the brain, but did not affect BP [239]. Studies with transgenic mice have further corroborated the detrimental effect of AT_1_R signalling in ischaemic stroke. Mice over-expressing human renin and angiotensinogen genes displayed higher levels of Ang II in the circulation and the brain and, as a result, worse neurological deficit following pMCAO compared to wild-type which was prevented with prior ARB treatment [240]. Moreover, over-expression of angiotensinogen resulted in a smaller area of salvageable brain tissue (penumbra) at one hour and larger infarct volume at 24 h following pMCAO, while knockout (KO) of the AT_1_R resulted in the opposite effect [241]. Although BP was affected by the overexpression of angiotensinogen (increased) or AT_1_R KO (decreased) which may have impacted the resultant infarct, the authors further demonstrated beneficial effects of AT_1_R KO on cell death in an oxygen glucose deprivation in vitro model suggesting BP independent effects [241]. Together, these results indicate a detrimental effect of AT_1_R signalling in the setting of ischaemic stroke through mechanisms including neuroinflammation, ROS generation, apoptosis and neurodegeneration, all of which are independent of BP yet clinically, systemic blockade of AT_1_R has not been shown to consistently improve stroke outcome or risk. Therefore, perhaps the counter-regulatory axis of the RAS provides an alternative therapeutic target and indeed, there is increasing evidence that activation of the counter-regulatory RAS receptors is beneficial for stroke outcome (Table 1 & Table 2).

**Table 1:** **Summary of studies utilising Mas receptor agonism in experimental stroke.** Abbreviations: AVE0991: Mas antagonist; BBB: blood brain barrier, BP: blood pressure, CBF: cerebral blood flow, COX-2: cyclooxygenase-2; DIZE: diminazene aceturate; eNOS: endothelial nitric oxide synthase ET-1: endothelin-1 injection, HPβCP-Ang-(1–7): Ang-(1–7) complexed with hydroxypropyl-β-cyclodextrins; ICV: intracerebroventricular, IL-1α/1β/6: interleukin-1α/1β/6; iNOS: inducible nitric oxide synthase; IP: intraperitoneal, IV: intravenous; MDA: malondialdehyde; NFκB: nuclear factor κ-light-chain-enhancer of activated B cells; NO: nitric oxide; NOX1: NADPH oxidase 1; SD: Sprague Dawley, SOD: superoxide dismutase; t/pMCAO: transient/permanent middle cerebral artery occlusion, TNF-α: tumor necrosis factor-α; VEGF: vascular endothelial growth factor.

**Animal.**	**Drug delivery dose, route & timing**	**Stroke model**	**Outcome**	**Mechanistic insight (if any)**	**Reference**
Male SD rats	Ang-(1–7) 1.1 nM/0.5 μL/h or DIZE 5 μg/ 5 μL/h (ACE2 activator) ICV minipump infusion 7 days prior & 3 days post	ET-1	Ang-(1–7): No effect BP No effect CBF ↓ infarct volume at 3 days ↑ neurological score & fine motor function at 3 days DIZE: ↓ BP with 7 days of treatment ↓ infarct volume at 3 days ↑ neurological score at 3 days	Positive stroke outcomes blocked by Mas inhibition. Ang-(1–7) attenuated stroke induced increase in iNOS expression	244
Male SD rats	Ang-(1–7) 1.11 nM/1 μL/h ICV minipump infusion 48 h prior & 24 h post	Filament pMCAO	No effect CBF ↓ infarct volume at 24 h ↑ neurological score at 24 h	Reduced oxidative stress (reduced MDA & increased SOD levels) Reduced NFκB activation Reduced expression of pro-inflammatory cytokines (TNF-α and IL-1β) and COX-2 Effects blocked by Mas inhibition but not AT_2_R inhibition	246
Male SD rats	Ang-(1–7) 1.1 nM/0.5 μL/h ICV minipump infusion 7 days prior & post until sacrifice	ET-1	↓ infarct volume at 24 h	Reduced expression of iNOS Blunting inflammatory response - reduced expression pro-inflammatory cytokines IL-1α & IL-6, & chemokine receptors CXCL12 & CXCR4, reduced expression of CD11b (marker of macrophage/microglial activation) In vitro evidence of reduction of NO production in glia, prevented by Mas inhibition	248
Male SD rats	Ang-(1–7) 1.1 nM/0.25 μL/h ICV minipump infusion 4 weeks prior to	Filament pMCAO	No effect BP prior to stroke ↑ peripheral CBF ↓ infarct volume at 24 h ↑ neurological score at 24 h	Increased eNOS activation, NO production, VEGF levels and angiogenesis Positive stroke outcomes and signalling effects abolished by Mas or eNOS inhibition	247
Male C57BL6/J mice, 8 week old	AVE0991 10 mg/kg or 20 mg/kg IP at reperfusion & 4 h post	Filament tMCAO 60 min	No effect CBF No effect neurological deficit, or locomotor and motor coordination at 24 h No effect infarct volume at 24 h	Reduced glucose deprivation induced neuronal death in vitro	252
Male SD rats, 9–13 weeks	HPβCP-Ang-(1–7) 125 μg/kg orally at 90 min, 4 h, 24 h & 48 h post hydroxypropyl-β-cyclodextrins,	ET-1	↓ infarct volume at 3 days ↑ neurological function at 3 days No effect CBF No effect BP	Not investigated	250
Male Wistar rats	Ang-(1–7) 1.1 nmol/μL/h ICV minipump infusion post-reperfusion	Filament tMCAO, 90 min	↑ tissue salvage at 7 days No effect infarct volume at 7 days No effect neurological score at 7 days No effect BP	Upregulation of NOX1 Prevention of ‘steal phenomenon’ in contralateral hemisphere perfusion No effect on BBB permeability, microglia activation or inflammatory markers	249

**Table 2:** **Summary of studies utilising AT**_**2**_**R agonism in experimental stroke.** Abbreviations: Akt: protein kinase B; Aβ: β-amyloid; BBB: blood brain barrier, BDNF: brain derived neurotrophic factor; BP: blood pressure, C21: compound 21, CBF: cerebral blood flow, CGP42112: peptide agonist of AT_2_R; eNOS: endothelial nitric oxide synthase; ET-1: endothelin-1 injection, GAP43: growth associated protein B; ICV: intracerebroventricular, IL-1β/10: interleukin 1β/10; iNOS: inducible nitric oxide synthase; IP: intraperitoneal, IV: intravenous; KO: knockout; MCP-1: monocyte chemoattractant protein-1; mTOR: mammalian target of rapamycin; PI3K: phosphoinositide-3-kinase; PPAR-γ: peroxisome proliferator-activated receptor-γ; SD: Sprague Dawley, SHR: spontaneously hypertensive rat, t/pMCAO: transient/permanent middle cerebral artery occlusion, TNF-α: tumor necrosis factor-α; tPA: tissue plasminogen activator; TrkB: tropomyosin receptor kinase B; ZO-1: zona occludens protein 1.

**Animal.**	**Drug delivery dose, route & timing**	**Stroke model**	**Outcome**	**Mechanistic insight (if any)**	**Reference**
Male SHR (15–16 week)	CGP42112, 0.1, 1 or 10 ng/kg/min, ICV minipump infusion 5 days prior +3 days post	ET-1, conscious	No effect BP. ↓ infarct volume at 3 days (1 & 10 ng doses). ↑ motor function at 1 & 3 days.	Increased neuronal survival Increased AT_2_R expression Reduced superoxide production All effects abolished by AT_2_R blockade	260
Male SHR	CGP42112 3 μg/kg/dose, ICV bolus at 6, 24, 48 & 72 h post stroke	ET-1, conscious	No effect BP. ↓ infarct volume at 3 days. ↑ motor function at 1 & 3 days.	Increased neuronal survival Reduced apoptosis Increased microglia activation All effects abolished by AT_2_R blockade	261
Male C57BL/6 mice (8–12 week)	CGP42112, 1 mg/kg, IP at reperfusion	Filament tMCAO, 30 min	↑ CBF at reperfusion. ↑ neurological score and motor function at 24 h. ↓ infarct volume at 24 h.	Reduced neuronal apoptosis in vitro prevented by AT_2_R blockade	192
Male SD (8 week)	C21, ICV 7.5 ng/μl/h minipump infusion for 7 days prior +3 days post *OR* IP 0.03 or 0.1 mg/kg prior to (2 h) and post(4, 24 & 48 h) *OR* IP 0.03 mg/kg 4, 24 & 48 h post	ET-1	↓ infarct volume with all delivery schemes at 3 days. ↑ neurological score with all delivery schemes at 3 days. IP delivery no effect on non-stroked BP or CBF.	Positive stroke outcome effects abolished by AT_2_R blockade Reduced expression of some pro-inflammatory genes: iNOS, CCL2 and CCR2 (chemokines)	264
Male SHR	C21, 50 ng/kg/min ICV minipump infusion 5 days prior & 3 days post *OR* 144 μg/kg ICV bolus at 6, 24, 48 & 72 h post	ET-1, conscious	No effect on BP ↓ infarct at 3 days with pre- and post delivery. ↑ motor deficit at 1 day (pre-treatment only).	Positive stroke outcome effects abolished by AT_2_R blockade Increased neuronal survival* Reduced apoptosis Increased microglia activation Suggestion of link between AT_2_R positive microglia and BDNF production Ex vivo vasorelaxation of cerebral arteries* *these effects also abolished by AT_*2*_*R blockade*	263
Male C57BL/6 mice (10–12 week)	C21, 10 μg/kg/day, IP after pMCAO, 24 h and daily thereafter	Electrocoagulation (pMCAO)	↓ infarct volume at days 1–5. ↑ neurological score at days 3–7. ↑ CBF at days 1 & 3 ↓ BBB disruption at 3 days.	AT_2_R KO mice had larger infarcts and infarcts not reduced by C21 Reduced superoxide production Reduced expression of proinflammatory cytokines TNF-α and MCP-1	262
Male Wistar rats	C21, 0.03 mg/kg IP at reperfusion	Filament tMCAO 90 min or 3 h	No effect BP ↓ infarct volume at 24 h ↑ neurological score & motor function at 1–7 days	Positive stroke outcome effects abolished by AT_2_R blockade Upregulation of AT_2_R and downregulation of AT_1_R expression Increased Akt and eNOS phosphorylation (pro-survival) Increased BDNF and IL-10 expression (neuroprotective) Decreased cleaved caspase-3 (apoptosis) Decreased nNOS, iNOS and nitrotyrosine (nitrative stress) Increased vascular density in vivo plus In vitro evidence of BDNF and AT_2_R dependent endothelial cell migration (pro-angiogenic)	267
Male C57BL/6 mice, WT or AT_2_R KO	C21 0.03 mg/kg IP 45 min post and daily thereafter	Filament tMCAO 30 min	No effect BP. No effect CBF. No effect infarct volume at 4 days. ↑ survival ↑ neurological score at day 1, 2 & 4	Increased levels of BDNF, its receptor TrkB and GAP43 (marker of neuronal outgrowth) Decreased neuronal apoptosis These effects plus improved neurological score were not seen with C21 treatment in AT_2_R KO mice	193
Male Wistar rats (8–12 week)	C21, 0.3 mg/kg/day, IP 6 h, 1, 2, 3, 4 & 5 days post	Filament pMCAO	↑ neurological score at days 3 & 4 ↓ infarct volume at days 5 & 21	Increased VEGF expression mediated by mTOR dependent mechanism Confirmed in vitro neuronal cultures to be mediated by PI3K/Akt/mTOR pathway	266
Male SD rats (12 weeks)	CGP42112 IP 1 mg/kg per day post (specific timing not stated)	Filament tMCAO 2 h	↓ infarct volume at 7 days	Increased AT_2_R expression Reduced expression of pro-inflammatory cytokines IL-1β & TNF-α. All effects abolished by AT_2_R blockade	79
Male SD (18–20 month)	C21 0.03 mg/kg IP 90 min, 1 & 2 days post	Filament tMCAO 45 min	↑ neurological score and motor function from 1 to 21 days. ↓ infarct volume at 3 weeks.	Not investigated	268
Male Wistar rats	C21 0.03 mg/kg IP at reperfusion	Filament tMCAO 3 h	↓ infarct volume at 21 h. ↑ neurological score and motor function at 21 h.	Positive outcomes partially mediated by IL-10 Reduced expression of pro-inflammatory TNF-α In vitro evidence of increased neuronal survival and reduced neuronal apoptosis	195
Male SHR (4 month)	C21 0.03 mg/kg/day IP at 2 h post	Filament tMCAO 60 min	No effect on BP. No effect neurological score and motor function at day 1 or 28. ↑ cognition at 21 days.	Reduced Aβ accumulation In vitro evidence of Aβ toxicity to neurons and endothelial cells Reduced sustained (30 days post stroke) microglial activation	265
Male SD (10–13 week)	C21 1.5 μg/kg intranasal delivery 1.5, 4, 24 & 48 h post	ET-1	No effect on BP. ↓ infarct volume at 3 days. ↑ neurological score at 1% 3 days.	Not investigated	271
Male C57BL6/J mice	C21 10 μg/kg/day IP for 2 weeks prior	Filament pMCAO	No effect on BP. ↓ infarct volume at 24 h. ↑ neurological score at 24 h. ↑ CBF at 24 h.	Positive outcomes partially mediated by PPAR-γ activation Reduced superoxide production and increased superoxide dismutase activity Increased expression of BBB stabilisation genes, occludin, claudin-5 and ZO-1	194
Male Wistar rats (8–10 week)	C21 0.01, 0.03 or 0.06 mg/kg IV at 3 h *OR* 0.01 mg/kg at 3 h & 0.04 mg/kg/day orally days 2–5 alone or in combination with tPA	Embolic	No effect on BP. ↑ neurological and motor function at days 3 or 5 (0.01 mg.kg dose). No effect infarct size. Trend towards ↑ cognition at 7 days. No effect when used in combination with tPA.	Not investigated	273
Male Wistar rats, healthy or diabetic (12–15 weeks)	C21 0.12 mg/kg orally at day 3 post	Filament tMCAO 60 min	↑ sensorimotor deficit at 1–8 weeks. ↑ cognition of diabetic rats at 1–8 weeks. Preserved brain volume at 8 weeks.	No effect on number of activated microglia but shift towards the M2 anti-inflammatory phenotype In vitro evidence suggests microglial may be independent of AT_2_R	270
Male Wistar (14 month)	C21 0.12 mg/kg orally at 24 h post and daily thereafter	Distal pMCAO, electrocoagulation	↓ weight loss No effect on neurological score or sensorimotor function at 28 days. ↑ cognition at 21 & 28 days.	Reduced Aβ accumulation No effect on BDNF concentration	272
Female Wistar rats (3–6 month)	C21 0.03 mg/kg/day IP at reperfusion followed by daily IP or 0.12 mg/kg/day orally	Filament tMCAO 1, 2 or 3 h	Trend towards ↓ infarct volume at 24 h and 14 days ↑ neurological score and trends with sensorimotor function at 24 h.	Trend towards increased PPAR-γ expression	269

## 6 Mas receptor and stroke

Experimental models have demonstrated that Mas receptor and ACE2 mRNA are upregulated in the brain during cerebral ischaemia and, as a result, brain and plasma levels of Ang-(1–7) are also increased, suggesting that the Mas receptor/ACE2/Ang-(1–7) axis plays a role in ischaemic injury [75]. Additionally, further studies indicated that neuronal-specific ACE2 overexpression reduces infarct volume and improves neurological score after pMCAO and this is mediated by the Mas receptor [242]. Although ACE2 overexpression did result in a reduction in BP, titrating the systemic pressure to a similar level to that of control mice with norepinephrine demonstrated that the beneficial effects were independent of BP [242]. Similarly, activation of ACE2 by systemic injections [243] or intracerebroventricular infusion [244] of the ACE2 activator, diminazene aceturate (DIZE), pre- or post-stroke resulted in beneficial effects without affecting BP or cerebral blood flow (CBF) but the effect was abolished with Mas receptor blockade [244]. Interestingly, these studies with ACE2 only utilised the Mas receptor blocker A779 and did not assess the effect of AT_2_R blockade despite knowledge that Ang-(1–9) is also produced by ACE2 [34]; although reports of Ang-(1–9) being an active peptide of the counter-regulatory RAS were only beginning to emerge around this time [47,245]. Use of the AT_2_R antagonist PD123,319 might indicate whether Ang-(1–9) is involved in any of the effects of ACE2 activation, although interpreting results might be challenging in this setting due to an ACE2 activator altering levels of many different angiotensin peptides. For example, Ang-(1–7) is also reported to utilise the AT_2_R [128] while PD123,319 is also known to block MrgD, the receptor for the alternative angiotensin peptide alamandine [48]. Coupled with the knowledge of heterodimerization of different angiotensin receptors [[51], [52], [53]] interpretation of data following use of PD123,319 might be complex.

Beneficial effects of delivery of Ang-(1–7), acting via Mas receptor and not AT_2_R, have also been demonstrated post-stroke in experimental models with both delivery prior to stroke induction [244,[246], [247], [248]] and after reperfusion [249,250] (Table 1). Time of delivery varied from 48 h [246], 7 days [244,248] to 4 weeks [247] prior to stroke, and continued to the study endpoint, typically 24 h [[246], [247], [248]] out to 3 days [244]. In those studies using post-stroke administration, treatment began as early as 90 min after occlusion (to coincide with reperfusion) [249] or at 90 min, 4/24/48 h after stroke [250]. Moreover, beyond the setting of ischaemic stroke, Ang-(1–7) treatment also improved survival and reduced haemorrhages in the stroke prone spontaneously hypertensive rat which is a model of spontaneous haemorrhagic stroke [251]. The non-peptide Mas receptor agonist, AVE 0991, has had less consistent results with improved outcome when delivered intranasally in a subarachnoid hemorrhage model attributed to reduced oxidative stress and reduced apoptosis [148] but no benefits observed with systemic delivery after stroke in an ischaemic stroke model [252]. In the latter study, the authors attribute the failure to dosing or timing issues considering they observed neuroprotective effects in an in vitro glucose deprivation model [252] and previous studies have demonstrated its ability to cross the blood brain barrier due to its hydrophobic nature [253].

The mechanisms behind Mas receptor neuroprotection in ischaemia reperfusion has been attributed to increased levels of bradykinin or the bradykinin receptors [154], reduced levels of ROS [246]; mediation of anti-inflammatory effects for example by inhibition of NFκB resulting in reduction of IL1β or TNFα [153,246,248]; improved BBB stability due to increased tight junction protein expression and reduced expression of MMP9 via TIMP1 [254]; reduced levels of iNOS [244]; and production of NO which, aside from vasodilatory effects, can also induce pro-angiogenic signalling [247] (Table 1). Although activation of eNOS resulting in NO production is a downstream signalling pathway of Mas in the brain [255] that can have a beneficial effect on stroke outcome [247], activation of iNOS may be detrimental to stroke outcome [256] and Ang-(1–7)/Mas signalling has demonstrated a reduction in iNOS expression [244,248].

These studies suggest an encouraging potential for Mas agonism as a neuroprotective treatment following ischaemic stroke, however the majority of promising results were obtained with intracerebral delivery of Ang-(1–7) (Table 1) with just one study using a more translationally relevant approach through oral administration [250]. Furthermore, all studies used only male healthy rodent animal models (SD or Wistar rats or C57BL6/J mice) and so co-morbidity and sex were not considered. That said, several stroke models have been studied including permanent and transient MCAO using endothelin-1 (ET-1) or the intraluminal filament models. Therefore, collectively, these studies fall short of many of the STAIR criteria for stroke studies [11] and there is much further work required before targeting this receptor could become a reality as a treatment for stroke patients.

## 7 AT_2_R and stroke

The importance of AT_2_R signalling in cerebral ischaemia was demonstrated with AT_2_R KO mice which displayed larger infarct volumes, worsened neurological score and reduced CBF, following pMCAO, than wild-type (WT) mice [257]. AT_2_R KO did not affect BP in these mice and inhibition of AT_1_R was not sufficient to rescue the effects of AT_2_R KO but rather demonstrates that the beneficial effects of AT_1_R inhibition in stroke is, in part, due to indirect AT_2_R stimulation [257]. Transient ischaemia also resulted in larger infarcts in AT_2_R KO mice compared to WT suggesting an important role in protection of IR injury [193]. Furthermore, numerous studies have shown, via different methods, that AT_2_R is upregulated following both permanent or transient MCAO which further suggests an upregulation as an endogenous protective response to ischaemia [77,258,259].

Agonism of AT_2_R has also demonstrated promising results in experimental stroke models (Table 2). Initial proof of concept studies utilised intracerebroventricular delivery of the peptide agonist of AT_2_R, CGP42112. Delivery five days prior to tMCAO significantly reduced infarct volume and improved motor deficits coupled with increased AT_2_R expression, improved neuronal survival and reduced ROS production in the infarct area, all of which were prevented by AT_2_R blockade [260]. More encouragingly, commencement of intracerebroventricular delivery of CGP42112 *after* tMCAO resulted in similar beneficial effects on infarct volume, motor deficit and neuronal survival as before, and also reduced apoptosis in the infarct and peri-infarct regions and increased microglia activation which may be a protective mechanism to remove cellular debris [261]. In both studies, CGP42112 had no effect on the BP of the spontaneously hypertensive rats (SHR) utilised. Systemic administration of CGP42112 at the commencement of reperfusion also had beneficial effects on infarct volume and functional outcome in a mouse stroke model suggesting the peptide does not need to be delivered directly to the brain to have an effect [192].

Most of the research on the benefit of AT_2_R agonism in stroke has been conducted using the non-peptide agonist, C21 (Table 2). Once daily intraperitoneal (IP) injections of C21 in the two weeks prior to pMCAO in WT mice and AT_2_R KO mice demonstrated that C21 reduced infarct volume in WT but not KO mice [262]. Further, C21 delivery only after pMCAO also improved neurological score and infarct volume out to seven days post-stroke, with improved CBF, reduced ROS, BBB permeability and proinflammatory cytokines three days after stroke [262]. These promising results were further corroborated in hypertensive rats, where continuous intracerebroventricular delivery of C21 five days prior to tMCAO, or as four bolus doses beginning 6 h after tMCAO, resulted in reduced infarct volume and improved neuronal survival which was prevented by AT_2_R blockade [263]. However only the pre-treatment arm and not the post-treatment arm also demonstrated improved motor deficit at 24 h and increased microglial activation [263]. It was further demonstrated that C21, via AT_2_R, induced vasorelaxation of basilar arteries in *ex vivo* myography experiments [263]. This may translate to an in vivo protective mechanism to improve CBF, although there was no effect of C21 on the systemic BP of the SHR [263]. Furthermore, both before and after stroke, a high proportion of BDNF positive cells were also positive for AT_2_R implying that AT_2_R signalling is involved in BDNF release [263]. Studies have since had conflicting results with regard to the effect of C21 on CBF with some demonstrating no effect [193,264] while others suggest improvement [194], but BDNF mRNA and protein levels have been shown to be increased with C21 treatment post-stroke and this effect is absent with AT_2_R KO [193]. Further studies demonstrated that IP delivery of C21 after stroke induction, whether permanent or transient, improved outcome after stroke including in co-morbid animals with hypertension or advanced age [193,195,[265], [266], [267], [268]] (Table 2). Additionally, AT_2_R signalling with C21 has demonstrated further translational potential with promising results achieved with post-stroke oral delivery in female rats [269] or in a type 2 diabetes animal model [270], or with an intranasal delivery approach resulting in high levels detectable in the cortex and striatum, and improved outcome following stroke [271].

Mechanisms behind the beneficial effects observed with C21 induced AT_2_R signalling in experimental stroke have been attributed to reduced proinflammatory cytokines [262,264], reduced apoptosis [193,265,267], reduced ROS and oxidative stress [194,262,267], increased VEGF production [266], increased BDNF production [193,267], a switch from the M1 to M2 microglia phenotype [270], BBB stabilisation [194,262], and increased angiogenesis [267] (Table 2). Some of these effects have been demonstrated to be dependent on IL-10 [195] or peroxisome proliferator-activated receptor-gamma (PPARγ) activation [194] (Table 2).

In addition, C21 AT_2_R signalling has also demonstrated reduced haemorrhagic transformation [267] and decreased β-amyloid (Aβ) deposition following stroke [265,272]. Indeed, Aβ deposition is implicated in cognitive impairment and C21 treatment has demonstrated beneficial effects on post-stroke cognitive impairment (PSCI) in both hypertensive [265] and aged rats [272], and in an embolic model of stroke [273]. Although results were promising in the embolic model, only C21 alone induced sensorimotor and cognitive improvements but not tPA alone or in combination with C21, suggesting translatability issues with the model considering tPA is the only clinically approved drug for stroke treatment [273]. Interestingly however, a recent clinical trial for the potentially neuroprotective compound NA-1 had similar results where benefits were seen only in patients who did not receive tPA [274]. This raises the possible potential for the use of drugs that offer benefits in the absence of tPA as a new treatment option for those patients who are ineligible for tPA treatment.

Further to the benefits to PSCI mentioned above, AT_2_R agonism with C21 has also demonstrated beneficial effects on cognition in animals models utilising chronic hypoperfusion [[275], [276], [277]], Aβ injections in the brain (AD model) [278] or in a type 2 diabetes model [279]. C21 produced increased CBF [276,278,279], reduced proinflammatory cytokines [276], increased levels of BDNF in the brain [279] and reduced Aβ deposition [277] in these models and although no effect on overall vascular remodelling was observed, one study did demonstrate increased size of the basilar artery which could be responsible for increased blood supply to the hippocampus [275]. Furthermore, combination of C21 with memantine, an NMDA antagonist, and therefore glutamate toxicity modulator, used in AD, resulted in even greater levels of BDNF in the brain but no additive effect on CBF or cognition was observed [279].

Interestingly, although the brain RAS plays an important role in regulation of systemic BP and AT_2_R signalling can oppose vasoconstrictive AT_1_R signalling, AT_2_R agonism by CGP4112 or C21 does not affect BP when the agonist is delivered before [262] or after [261,267] MCAO either by systemic [264], intracerebral [260] or intranasal [271] delivery nor after embolic stroke [273]. However, contrasting studies have been reported a marked hypotensive response following central C21 administration into conscious normo- [280,281] or hypertensive [281] rats. Therefore, given the uncertainty surrounding BP lowering in acute ischaemic stroke care, the lack of BP effect by AT_2_R agonism in stroke is encouraging and reassuring but should be considered cautiously.

Clearly there is strong evidence to support the role of protective effects of AT_2_R signalling in the brain and the potential of targeting this receptor as a treatment for ischaemic stroke and possibly PSCI. Collectively, these studies demonstrate consideration of many of the STAIR guidelines criteria for preclinical stroke studies [11] arguably placing this potential novel therapeutic, C21, ahead of other strategies to target the counter regulatory axis of the RAS in the setting of stroke. Furthermore, recently C21 was shown to be safe and well-tolerated when administered orally in healthy adult male volunteers [282] and further ongoing trials with C21 in Raynaud's phenomenon (ClinicalTrials.gov Identifier: NCT04388176), idiopathic pulmonary fibrosis (ClinicalTrials.gov Identifier: NCT04533022) and COVID-19 (ClinicalTrials.gov Identifier: NCT04452435) will provide further evidence on its safety in human subjects.

## 8 Conclusion

Cellular signalling via the Mas receptor and AT_2_R of the counter regulatory axis of the RAS has been well established and provides multiple mechanisms to oppose negative signalling effects of the AT_1_R within the cardiovascular system. Additionally, many of these signalling mechanisms have been confirmed within the brain and could potentially aid neuroprotection and brain repair following stroke. Indeed, preclinical stroke studies utilising agonism of the counter regulatory axis of the RAS demonstrate consistently encouraging results across several experimental and animal models, although often the cellular signalling mechanism mediating the beneficial effect is not confirmed. Encouragingly, many of these studies have used dosing protocols which would align with the window of therapeutic intervention afforded through existing stroke treatments, tPA or intra-arterial thrombectomy. Further well designed preclinical studies, for example utilising mixed sex cohorts with *co*- or multi-morbidities and considering the polypharmacy associated with stroke patients may see progress in the targeting of the brain counter regulatory RAS axis in stroke patients.

## Declaration of Competing Interest

The authors declare that they have no known competing financial interests or personal relationships that could have appeared to influence the work reported in this paper.
